# Population genetics of the Manila clam (*Ruditapes philippinarum)* in East Asia

**DOI:** 10.1038/s41598-020-78923-w

**Published:** 2020-12-14

**Authors:** Yue Tan, Lei Fang, Ming Qiu, Zhongming Huo, Xiwu Yan

**Affiliations:** 1 Engineering Research Center of Shellfish Culture and Breeding in Liaoning Province, No.52, Hei Shi jiao Street, Dalian City, 116023 China; 2 College of Fisheries and Life Science, Dalian Ocean University, No.52, Hei Shi jiao Street, Dalian City, 116023 China; 3 College of Marine Science and Environment, Dalian Ocean University, No.52, Hei Shi jiao Street, Dalian City, 116023 China

**Keywords:** Evolutionary genetics, Genetic markers

## Abstract

The Manila clam *Ruditapes philippinarum* is the world’s second most important bivalve mollusk commercially farmed, whose indigenous populations are mainly distributed in the coastal areas of East Asia. However, with the development of commercialization, mixture of populations and loss of local germplasm have become prominent problems. Here, genetic differentiation of seven Manila clam populations from East Asia was investigated through analyzing the polymorphism of the mitochondrial cytochrome C oxidase subunit I (*COI*) gene as well as 20 simple sequence repeat (SSR) molecular loci. In total, 40 haplotypes were identified, among which 31 were unique. Moreover, two main haplotypes were detected with several radiating derived haplotypes. Populations in Japan-North Korea shared haplotype Hap_31, and populations in China shared haplotype Hap_7, suggesting that the natural geographical isolation of the Yangtze River and the Yalu River might have divided the East Asian indigenous populations into three groups, which were located in South China, North China, and Japan-North Korea, respectively. The Aquaculture breeding activities from South to North in China might have promoted gene exchange among Manila clam populations. Population in Laizhou had the highest genetic diversity and therefore could be an excellent germplasm source.

## Introduction

The Manila clam *Ruditapes philippinarum* (synonym *Venerupis philippinarum*)^1^ is a eurythermal and euryhaline species. Indigenous populations are distributed along the coasts of the Pacific to the Indian Ocean. In 1936, the Manila clam was introduced from Japan to British Columbia and subsequently from North America to the European coasts of France, Britain, Italy, Spain, and Turkey. Because it is characterized by long larval floating time (2–3 weeks), widespread distribution, and strong environmental adaptability, it is now widely distributed and has become the world’s second most important commercially cultured bivalve mollusk^1–5^.

The conditions along the coast of East Asia are favorable for genetic studies of the Manila clam. First, the extensive coastline of East Asia, especially the area near the Yangtze River delta, significantly contributes to the biological distribution pattern of the Manila clam in coastal areas. Natural barriers provide unique geographical conditions that are key to investigating the evolution of the indigenous Manila clam population and its geographical and historical patterns. Second, the annual production of Chinese Manila clams is about 3 million tons, accounting for > 90% of the world’ production. This species exhibits numerous genetic variations in growth, temperature and salinity tolerance, shell color, and shell shape^2^. China is the world’s largest Manila clam germplasm resource, which provides an important genetic basis for studies of population genetic differentiation and genetic diversity of Manila clams. Third, in recent years the northern coastal ecosystems of China have been impacted by economic development, frequent commercial activities, land reclamation from the sea, and artificial breeding of Manila clams using seedlings from southern China. These changes have resulted in mixing of the Manila clam populations and loss of the indigenous germplasm, which have prompted studies of Manila clam population gene exchange and genetic structure changes.

The genetic diversity of the Manila clam has been studied extensively. For example, Cordero et al.^6^ combined the mitochondrial cytochrome C oxidase subunit I (COI) gene sequence and/or the simple sequence repeat (SSR) gene marker to assess the genetic basis of the Manila clam population introduced from Japan to the North American west coast in 1936. Chiesa et al.^7^ analyzed the introduction history of European Manila clams using the COI gene sequence. Sekine^8^ assessed the genetic differentiation between the Japanese and Chinese Manila clam populations through COI gene analysis and found that the Japanese population can be divided into two subpopulations and the Chinese population can be divided into two subpopulations.

The genetic structure of the populations in Beihai, Lianjiang, Laizhou, Yingkou, and Tianjin in China as well as the populations in Sinuiju (North Korea) and Hokkaido (Japan) are important wild seed production areas. In this study, we used the mitochondrial COI gene^9^ and microsatellite loci to analyze the genetic structure of these seven Manila clam populations in East Asia. Our results provide a hints for the conservation and utilization of Manila clam germplasm resources.

## Materials and methods

### Sampling

**Figure 1:**
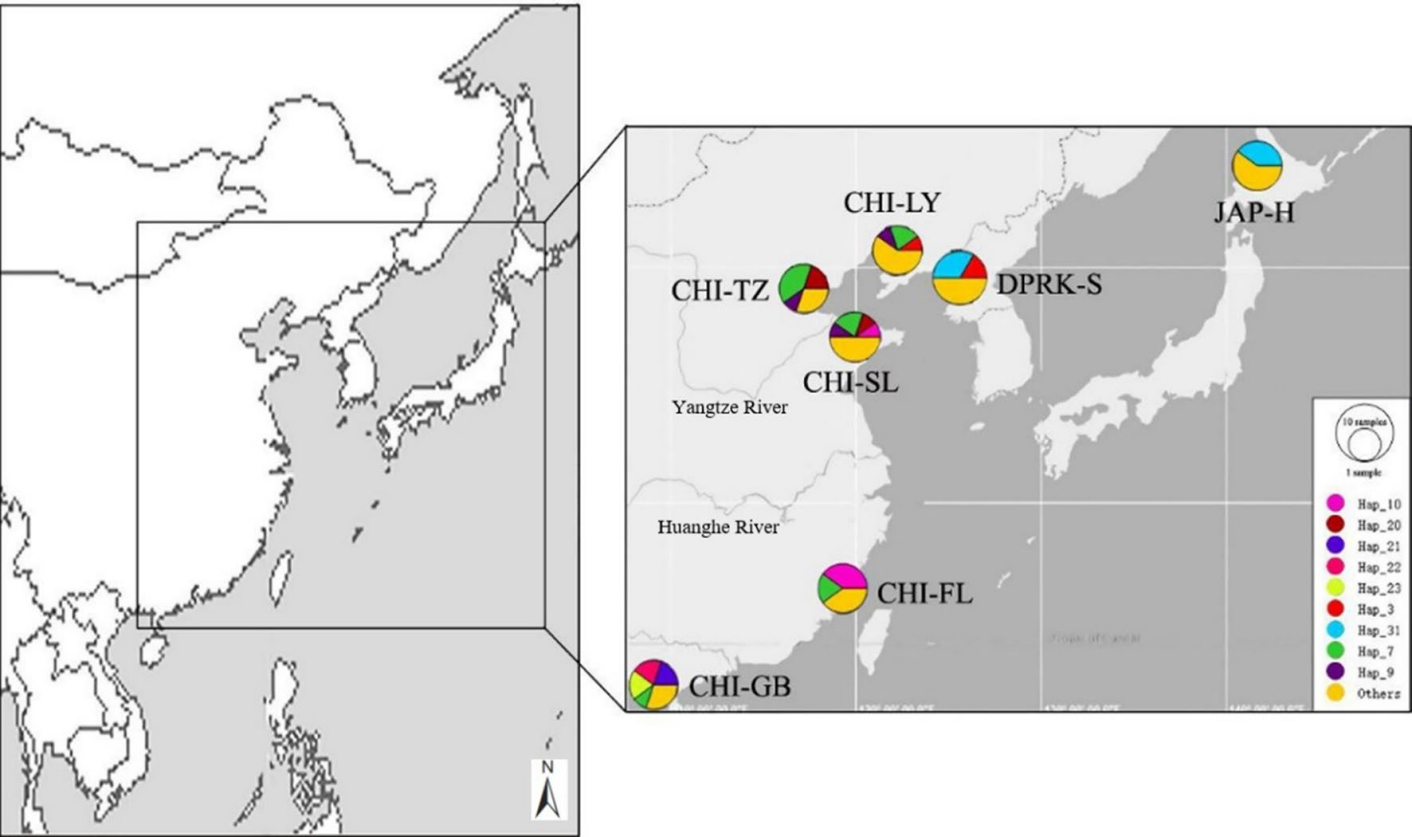
Frequency distribution of the COI haplotypes found among Manila clam populations. Pie charts show the distribution of COI haplotypes in the seven populations. Maps have been acquired from Google Maps. Map data ©2017 Google, SK Telecom, Zenrin. Pie charts were drawn using Popart^13,14^. Note, Sampling locations, CHI-LY (Ying Kou, China); CHI-FL (Lian Jiang, China); CHI-TZ (Zui Dong, Ch ina); CHI-SL (Lai Zhou, China); CHI-GB (Bei Hai, China); JAP-H (Hokkaido, Japan); DPRK-S (Sinuiju, North Korea).

To investigate the genetic diversity and genetic structure of Manila clams in the context of degenerated germplasm resources and contaminated native populations, seven populations were selected as the experimental materials, including three northern China populations, two southern China populations and two Japan-and-Korean populations (Table 1; Fig. 1). To eunsure random sampling, clams from all the seven populations were manually collected from the sedimental bottom. The foot and adductor muscle were removed from fresh specimens and preserved in 90% ethanol until DNA extraction. Studies of marine bivalves often report values of Observed Heterozygosity (HO) which were lower than those expected under Hardy–Weinberg equilibrium^10^, which might be due to inbreeding, natural selection, mutation, null alleles, gene flow, genetic drift^11^. O'Connell and Wright^12^ suggested that a minimum sample size of 50 individuals per population should be considered for loci with between 5 and 10 alleles. In this study, 50 individuals were sampled for each sample site to ensure a sufficient sample size for an accurate assay. The sampled clams were then dissected in the laboratory and used for subsequent DNA extraction. For each population, ten individuals were used for COI gene sequencing and fifty individuals were used for microsatellite analyses.

**Table 1:** Sample details for the seven populations of the Manila clam.

Sampling area	Population	Abbreviation	No. individuals (COI)	No. individuals (Microsatellite)	Sampling time	Latitude (N)	Longitude (E)
North China	Yingkou	CHI-LY	10	50	2016.06	40°40′16″	122°09′02″
	Zuidong	CHI-TZ	10	50	2016.07	38°57′48″	117°43′45″
	Laizhou	CHI-SL	10	50	2016.07	37°24′12	119°56′44″
South China	Lianjiang	CHI-FL	10	50	2016.08	26°11′04″	119°37′50″
	Beihai	CHI-GB			2016.05	21°28′24″	109°07′09″
Japan-North Korea	Hokkaido	JAP-H			2017.09	39°84′32″	124°81′34″
	Sinuiju	DPRK-S	10	50	2017.08	44°43′21″	141°67′56″

### DNA extraction

The phenomenon of DUI (Doubly Uniparental Inheritance) has been reported in Manila clam^9^. In DUI, male Manila clam has two mitochondrial genomes (one each from the male (M) and female (F) parent), and the F-type mitochondrial genome is inherited from the female in somatic muscle tissue and the M-type mitochondrial genome is inherited from the male in the gonad. The nucleotide sequence of M-type mitochondrial DNA is up to 30% different from the F-type mitochondrial DNA. Maternally inherited F-type mitochondrial DNA is the best choice for studying systematic classification and population inheritance. Therefore, in this study, foot and adductor muscle tissues were used for population analysis of the Manila clam F-type mitochondrial DNA COI. Genomic DNA of each specimen was extracted from muscle tissues using the Marine Shellfish DNA Extraction Kit (Tiangen, Beijing, China) and stored at − 20 °C. Total DNA was used for both mitochondrial DNA and microsatellite analysis.

### Mitochondrial DNA amplification and sequencing

The universal forward primer COI-F (5′-GGTCAACAAATCATAAAGATATTGG-3′) and universal reverse primer COI-R (5′-TAAACTTCAGGGTGACCAAAAAATCA-3′)^15^ were used to amplify a fragment of the COI gene. For each sample, polymerase chain reaction (PCR) was performed in a final volume of 50 μL containing 16 μL of sterile deionized water, 25 μL of Mix containing Takara *Taq* enzyme, buffer, and dNTP (Takara, Dalian, China), 2 μL of each primer (20 m mol L^−1^), and 5 μL of DNA template (100 ng μL^−1^). The amplification reaction was carried out in a PTC-100 thermal cycler according to the following temperature profile: 5 min initial denaturation at 94 °C; 34 cycles of 94 °C for 30 s of denaturation, 52 °C for 30 s of annealing, 72 °C for 30 s of extension, a final extension of 7 min at 72 °C, and a final hold at 4 °C. All verified PCR products were purified using a Takara MiniBEST Agarose Gel DNA Extraction Kit and then subjected to DNA sequencing in both directions with corresponding PCR primers using an ABI 3730xl DNA Analyzer sequencing system (Sangon Biological Engineering Company, Dalian, China).

### Microsatellite selection and genotyping

According to the transcriptome data of *R. philippinarum* (data not published), 20 pairs of primers (Table S1) were designed to amplify various microsatellit loci. For each population, ten individuals were randomly selected for PCR amplification using the above mentioned primer pairs. For those primer pairs generating convincible products, another ten individuals from each population were randomly selected for a second round of PCR analysis to verify the amplification. Through the two rounds of screening, twenty pairs of primers whose products showed sound polymorphism (with 3 to 12 polymorphic loci) were selected to analyze the genetic structure of different Manila clam populations. PCR conditions were optimized using different concentrations of primers, dNTPs, Mg^2+^, template DNA, and Taq polymerase as well as different annealing temperatures. Based on the optimization experiments, PCR was carried out in a volume containing 1 × PCR buffer, 0.25 U Taq DNA polymerase, 1 mM of each primer set, 0.2 mM dNTP mix, 1.5 mM MgCl_2_ (Takara), and about 100 ng of template DNA in a volume of 10 μL. The amplification reaction was carried out in the PTC-100 thermal cycler using the following temperature profile: 5 min initial denaturation at 94 °C; 34 cycles of 94 °C for 30 s of denaturation, 50–62 °C for 30 s of annealing, 72 °C for 30 s of extension, a final extension of 7 min at 72 °C, and a final hold at 4 °C. Amplification products were separated by 8% denaturing polyacrylamide gel and visualized by silver staining. A 10 bp DNA ladder (Takara) was used as the molecular size marker.

### Bioinformatic analysis

DNA sequences obtained were edited and aligned using DNAMAN (Lynnon BiSoft) to obtain a consensus sequence. The haplotype diversity (Hd), number of haplotypes (h), nucleotide diversity (π), and average number of nucleotide differences (K) were computed using DNASP 5.10.01^16^. Arlequin v3.5^17^ was used to estimate the population average pairwise differences among populations and pairwise genetic distance (*F*_ST_). Haplotype network analysis was performed through POPART^13,18,19^. The confidence threshold for the Manila clam sequence is 95%, and it was used to test whether haplotypes form a single network separate from that of congeneric species^20,21^. Data were converted into a nexfile using DNASP software, and we mapped the median-joining (MJ) network using POPART v1.7 (http://www.http://popart.otago.ac.nz/index.shtml) to track the genetic relationship among the identified haplotypes and other breeds from different regions. A MJ network^13^ was then constructed using POPART v1.7 for Manila clam haplotypes and outpopulations. Additionally, *F*_*ST*_ values were calculated using MEGA v7^22^ to assess the inter-population relationships for the genetic and geographic distance matrices.

Beast v2.6.2 software^23^ and Tracerv1.4^24^ software were used to estimate the divergence time between branches with constant rate based on the degree of divergence between gene sequences and molecular clocks, and the occurrence times of other nodes on the phylogenetic tree also were calculated. The Bayesian Skyline Plot (BSP) method is based on coalescent theory and was used to quantify the relationship between gene sequence pedigree and population geographic history to infer the origin time of related populations and the divergence time of different populations.

STRUCTURE v2.3.3^25^ software was used for population genetic structure analysis, with K value set to 2–9. The iteration parameters of Burn in Period and Markov Chain Monte Carlo were 100,000 and 50,000, respectively. Structure Harvester (http://taylor0.biology.ucla.edu/struct_harvest/) was used to determine the score of the ΔK value and the optimal number of gene banks (K value). CLUMPP v1.1.2 software^26^ was used to calculate the individual membership coefficient (Q value) based on the optimal K value, and DISTRUCT v1.1 software^27^ was used to visualize the population genetic structure.

### Microsatellite genotyping and data analysis

For the SSR analysis, each individual electrophoresis band represents a specified locus, and the molecular weights of the loci were determined according to their location. According to the banding migration distance, we used the letters a–z to record each individual’s genotype. Number of alleles per locus (*N*_*A*_), allelic richness (*A*_*R*_), size in base pairs of alleles, expected heterozygosity (*H*_*E*_), observed heterozygosity (*H*_*O*_), Shannon’s Information index (*I*) and inbreeding coefficient (*F*is) were estimated using POPGENE32 software^28^.

The polymorphism information content (PIC) is generally used to measure the degree of microsatellite marker DNA variation and as an indicator of *A*_*R*_ (i.e., diversity)^29^. PIC is calculated as follows:

$$PIC=1-\sum_{i=1}^{n}{P}_{i}^{2}-\sum_{i=1}^{n-1}\sum_{j=i+1}^{n}{2P}_{i}^{2}{P}_{j}^{2}$$

where n denotes the number of alleles at the microsatellite and P_i_ and P_j_ denote the i^th^ and j^th^ allele frequency, respectively. When the PIC value in a population is > 0.5, the locus is highly polymorphic. When the PIC value is between 0.25 and 0.5, the locus is moderately polymorphic, and when it is < 0.25, polymorphism of the locus is low.

### Ethical approval

The authors followed all applicable international, national, and/or institutional guidelines for the care and use of animals.

### Sampling and field studies

All necessary permits for sampling and observational 285 field studies have been obtained by the authors from the competent authorities and are mentioned in the acknowledgements, if applicable.

### Experimental protocols approved

The experimental protocols were approved by the institutional and licensing committee by including a statement in the methods section.

## Results

### Mitochondrial DNA

**Figure 2:**
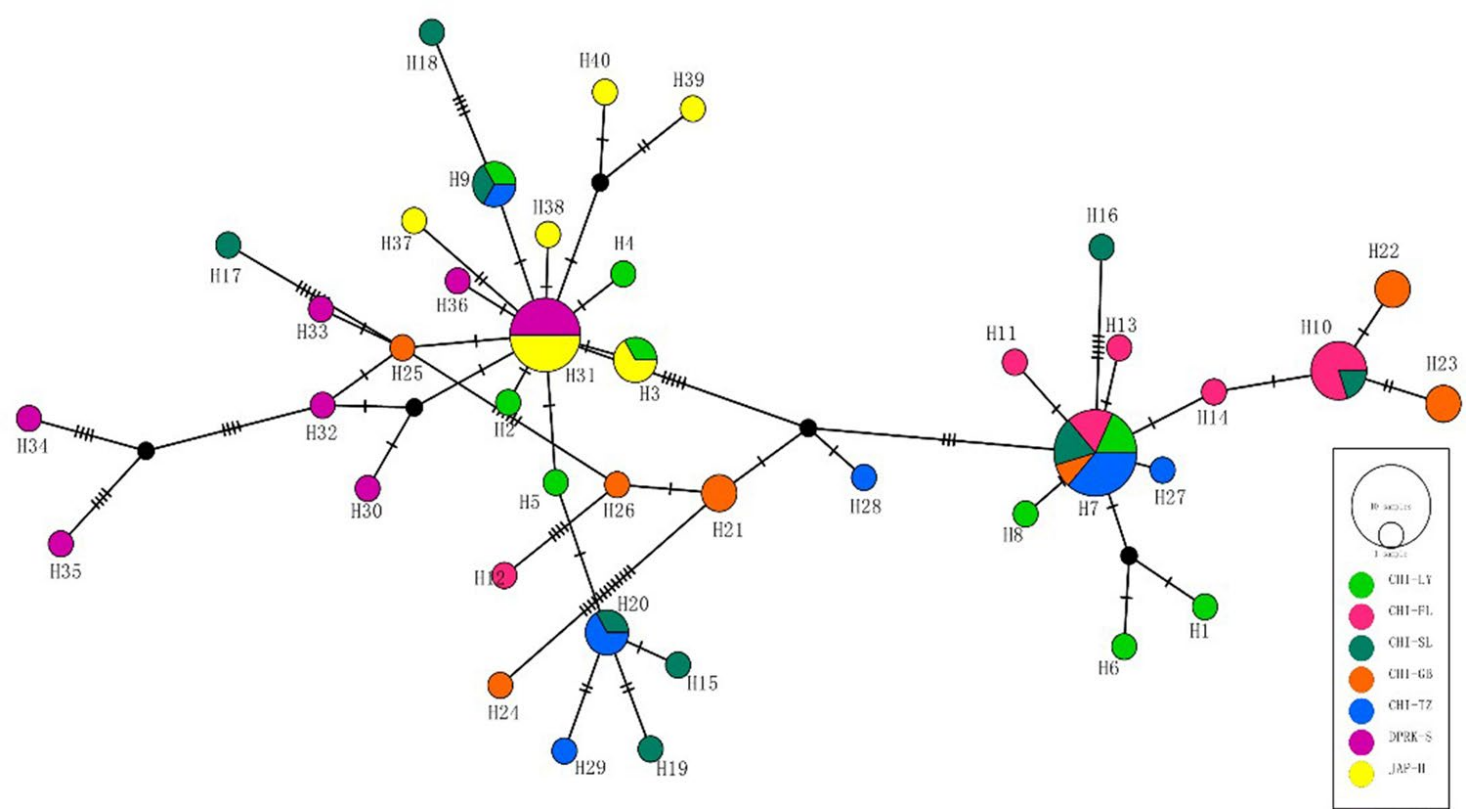
The phylogenetic network otained using the MJ algorithm shows the evolutionary relationships of the COI sequences in this study^13^. The color of the sector indicates the haplotype frequency in the seven populations studied. The size of the circle is proportional to the haplotype frequency.

A 680 base pair sequence of the mitochondrial COI gene from 70 individuals was successfully obtained (accession no: MW093623-MW093692). A total of 40 haplotypes were identified, 31 of which were unique (Table S2). Nine closely related COI haplotypes were clearly present among the East Asian populations (Fig. 1), and unique haplotypes were detected as well (i.e., in South and North China and in Japan-North Korea). Hap_30 was identified only in the northern populations (Hokkaido (JAP-H) and Sinuiju (DPRK-S)). Hap_3, Hap_9, and Hap_20 were found in northern China (Tianjin (CHI-TZ), Yingkou (CHI-YK), and LaizhouCHI-SL) but were absent from southern China (CHI-GB and CHI-FL). Hap_21, Hap_22, and Hap_3 were only observed in the North China Sea population of CHI-GB. Hap_23, Hap22, Hap_7, and Hap_10 found in CHI-GB and CHI-FL were not observed in Hokkaido (JAP-H) and Sinuiju (DPRK-S) (Fig. 2).

All the individual haplotypes could be categorized into two clades. The haplotypes Hap_31 and Hap_7 were the two centers, which were surrounded by the other haplotypes in a radial manner. The clade centering Hap_31 was found in the Hokkaido (JAP-H) and Sinuiju (DPRK-S) populations only, whereas the clade centering Hap_7 was found in the five populations located in China.

**Figure 3:**
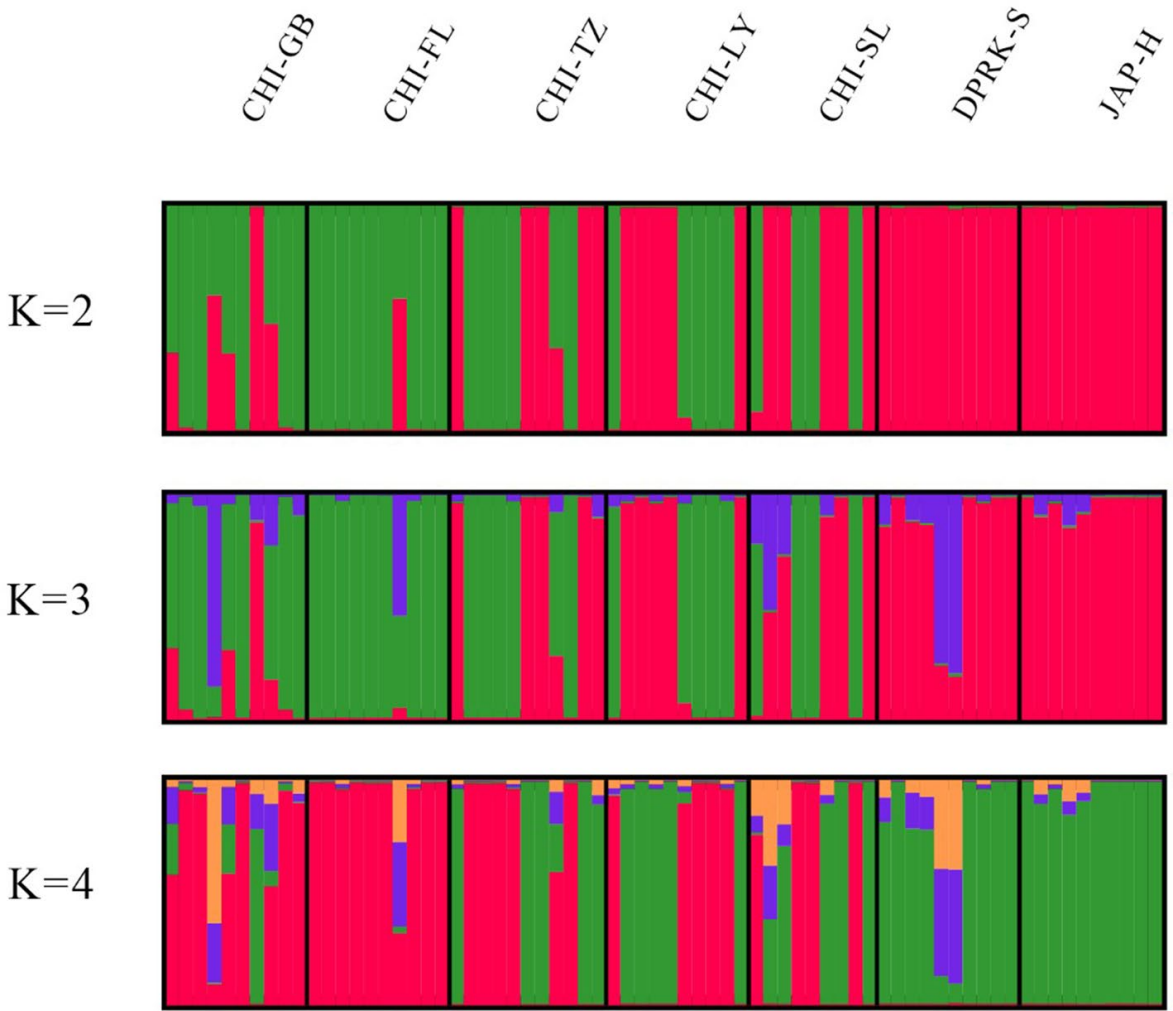
Genetic variability in Manila clam populations. (A) Haplotype diversity (± SD) estimated from the whole set of COI sequences. (B) Nucleotide diversity (± SD) estimated from the whole set of COI sequences.

Our analyses revealed high levels of Hd and π. Hd ranged from 0.9778 to 0.8444 (Fig. 3A), and π ranged from 0.01446 to 0.00279 (Fig. 3B). The highest Hd, π and nucleotide differences (K) values were found in the CHI-SL samples (Fig. 3 and Table S3).

**Figure 4:**
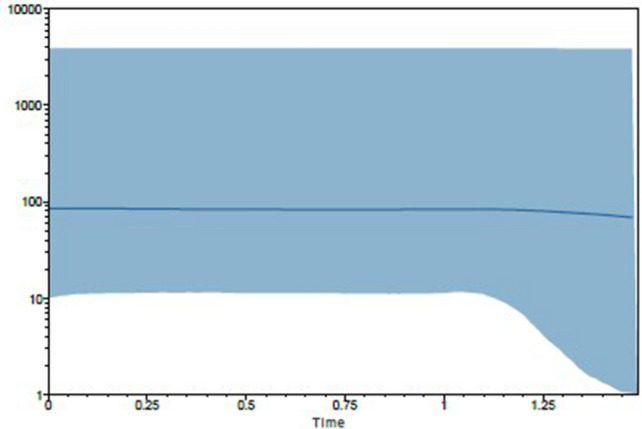
Bayesian model-based cluster analysis of individual genotypes at COI locus in 7 populations of Manila clam. Estimated membership fractions for each individual and population are shown for K = 2, K = 3 and K = 4 clusters. Cluster1 (Red), Cluster2 (Green), Cluster 3 (purple).

Figure 4 shows the results of Bayesian model-based clustering (BMBC) of genotypes. The Pritchard method showed a most probable K = 3 (Fig. S1). Cluster 1 was most abundant in JAP-H and DPRK-S and almost absent in CHI-FL and CHI-GB. Cluster 2 was most abundant in CHI-FL and CHI-GB and almost absent in JAP-H and DPRK-S. Clusters 1 and 2 were present in CHI-TZ, CHI-LZ, and CHI-SL (Fig. 4).

Data sets were further used to calculate population pairwise *F*_*ST*_ to compare the seven populations (Table 2). The genetic differentiation between these populations is relatively different, with *F*_*ST*_ value ranging from − 0.04928 to 0.75000 (mean *F*_*ST*_, 0.24475). However, the values differed significantly in pairwise comparisons between JAP-H or DPRK-S and the other populations.

**Table 2:** Results of F-statistics analysis of seven regions for COI.

	CHI-LY	CHI-FL	CHI-SL	CHI-GB	CHI-TZ	DPRK-S	JAP-H
CHI-LY	0.00000						
CHI-FL	0.29353***	0.00000					
CHI-SL	− 0.03299	0.29698***	0.00000				
CHI-GB	0.11306	0.05163	0.12455*	0.00000			
CHI-TZ	− 0.04928	0.24162*	− 0.03339	0.07863	0.00000		
DPRK-S	0.26577***	0.66110***	0.15821***	0.43351***	0.33035*	0.00000	
JAP-H	0.29784***	0.75000***	0.17143*	0.49854***	0.38047*	0.10811*	0.00000

*P < 0.05; **P < 0.01; ***P < 0.001.

**Figure 5:**
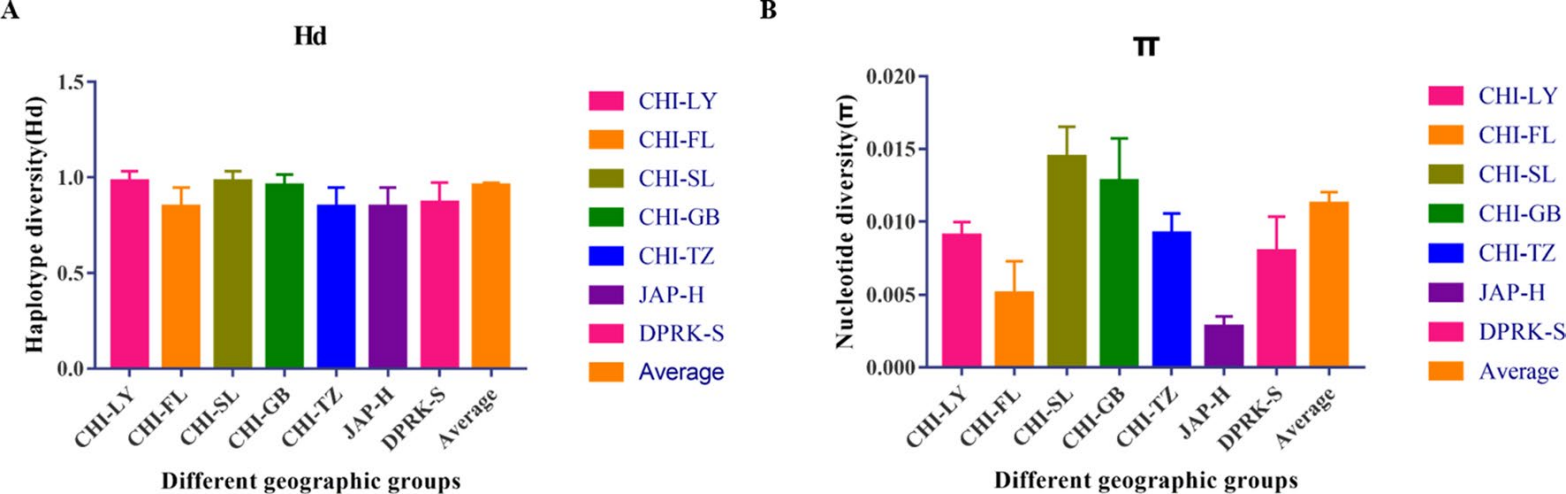
Bayesian skyline plots showing changes through time of *NeT* with uncorrected substitution rate (*R* = 1). (Ne = effective population size; T = generation time with uncorrected substitution rate (R-1) based on mtDNA control region sequences of all populations).

DNASP software was used to analyze the distribution of population mismatch, and the distribution curves of the mismatch were bimodal or multi-peak (Fig. S2). Population mismatch results indicated that population expansion of Manila clams did not occur recently. However, the BSP method showed that the Manila clam population underwent population expansion 12,500 years ago, but it became for stable 11,000 years (Fig. 5).

### Microsatellites

All 20 microsatellite loci were polymorphic in all Manila clam populations studied, and the levels of polymorphism varied among loci. In total, 144 alleles were detected at the 20 microsatellite loci analyzed. RPg15608 with 12 alleles was the most polymorphic microsatellite, whereas RPg15130, RPg16260, RPg16876, and RPg16965 were the least variable with only five alleles. Table S4 provides the genotype data for the seven Manila clam populations, including the parameters of *N*_*A*_, *A*_*R*_, *H*_*O*_, and *H*_*E*_, which were used to assess the level of genetic diversity. The *N*_*A*_ at the genomic SSRs ranged from 3 to 12 (mean *N*_*A*_ 5.3929). The *A*_*R*_ at the genomic SSRs ranged from 3.4458 to 4.219 (mean *A*_*R*_ 3.8919). The Shannon’s Information index (*I*) at the genomic SSRs ranged from 1.321 to 1.5331 (mean (*I*) 1.4322). The *H*_*E*_ per locus for genomic SSRs ranged from 0.7011 to 0.7542 (mean *H*_*E*_ 0.7276). The highest *H*_*E*_ value was detected for RPg15608 (0.8416), and the lowest value was found for RPg16975 (0.6633).

At the population level, the average *H*_*O*_ ranged from 0.2803 to 0.5220, and the average *H*_*E*_ ranged from 0.7011 to 0.7542. The highest *H*_*E*_ value was detected in population CHI-SL (0.5220) (Table S4). The *A*_*R*_ ranged from 1.7864 to 8.3333 for each sample. Of all loci, the highest value (*A*_R_) was found in the CHI-SL population (5.70). There was no significant difference in the average *A*_*R*_ among seven populations. RPg15246, RPg15130, and RPg16975 showed slightly negative *F*is values (Table S4).

The PIC results showed that there were 20 polymorphic loci in this study, and the PIC value of polymorphic microsatellite loci was between 0.39222 and 0.86806 (Table S5). The PIC of RPg18070 (0.86806) was the largest among the Manila clam populations in Laizhou. The 20 polymorphic microsatellite loci in the Yingkou, Lianjiang, and Laizhou populations were all highly polymorphic, and the percentage of highly polymorphic loci was 100%. In the Beihai and Hokkaido populations, 19 out of 20 polymorphic microsatellite loci were highly polymorphic and the other locus was moderately polymorphic; the percentage of highly polymorphic loci was 95%. In the Zuidong population, 17 loci were highly polymorphic, 3 loci were moderately polymorphic, and the percentage of highly polymorphic loci was 85%. Genetic linkage analysis suggests that microsatellite markers with PIC > 0.7 are the most ideal selection markers^29^. Based on the mean PIC, Laizhou population (0.70755) had the highest genetic diversity among the seven populations.

## Discussion

### Genetic differentiation of three East Asian Manila clam populations

Based on the comparison of haplotypes of COI sequences of Manila clam populations from different geographic areas using haplotype networks, BMBC, and the *F*_ST_, the coastal populations in East Asia can be grouped into South China, North China, and Japan-North Korea populations. Additionally, we detected endemic haplotypes in South and North China and in Japan-North Korea.

We found two main haplotypes in the seven populations studied, and the other haplotypes were distributed radially around these two haplotypes. The Japan-North Korea populations shared Hap_31, and the five populations in China shared Hap_7. Most of the other haplotypes were detected only in an individual area. This phenomenon, which is related in part to frequent overharvesting of Manila clams in coastal areas, also has been detected in the population genetic structure of other invertebrates^30^.

BMBC analysis based on COI supported the haplotype network tree results. This analysis with k = 3 showed clear differentiation between Manila clam populations in southern China (i.e., Lianjiang and Beihai) and northern Japan-North Korea. Cluster 1 was most common in the Hokkaido and Sinuiju samples but was rare in South China. Similarly, Cluster 2 was most abundant in South China but was almost completely absent from the Japan and North Korea Manila clam populations.

The *F*_ST_ results also revealed obvious genetic differentiation between the Japan-North Korean populations and the South and North China Manila clam populations. This is likely attributable to the natural barrier formed by the Yangtze River delta, which blocks gene exchange of natural populations of Manila clams in South and East Asia. After tens of thousands of years of isolation and population evolution, Manila clams have formed distinctive southern and northern populations, and fresh water flow from the Yalu River further divided the Northeast Asian Manila clam populations into the North China and the Japan-North Korean Manila clam populations. Similar results were observed in previous studies. For example, Cordero et al.^6^ divided the Chinese and Japanese Manila clam populations into two independent clusters. Kitada et al.^31^ compared the COI sequence and shell morphological traits between the Japanese and the southern Chinese Manila clam populations and found that the shell morphology and radial ribs of the two populations were significantly different.

The larvae of Manila clam have a long floating time (2–3 weeks), widespread distribution, and strong environmental adaptability, facilitating gene flow within regions and genetic mixing of populations. Therefore, within a certain space, gene flow can homogenize the Manila clams^32^.We found evidence of genetic exchange among different populations in the southern and northern Manila clam populations in China because of the influence of coastal currents of the Bohai Sea and the South China Sea^33^. Therefore, genetic differentiation of the three Manila clam populations from Tianjin, Yingkou, and Laizhou in North China from the populations in Guangxi and Fujian in South China was insignificant.

The level of genetic structuring among populations is the result of the balance between genetic drift, which promotes differentiation, and gene flow, which promotes homogenization of genetic differences. Gene flow among populations in South and North China might follow the isolation-by-distance model, as the Manila clam is a species with a demand for limited living space, thus, they show no large range movement from birth to death. Each population is spatially continuous. The range of random mating between individuals is limited, the frequency of genes differs little between adjacent populations, and there is a connection between geographic location and genetic distance between different populations. However, we detected clear genetic differentiation between the Japanese and Korean Manila clam populations in Hokkaido and Sinuiju. Results of COI haplotype genetic diversity and DNA diversity analyses suggested that the Hd of the Hokkaido population was high (0.8440) and that π was low (0.00279), which was likely due to rapid population expansion of small populations after experiencing the bottleneck effect. One possible explanation for this pattern is that the Japanese population has experienced overfishing and is located in an island and therefore does not communicate easily with other populations in the Sea of Japan. This may be one of the reasons for the obvious genetic differentiation between the Hokkaido and Sinuiju populations.

### Gene exchange between North and South China Manila clam populations

The haplotype network results for the Chinese populations showed that Hap_7 had the highest frequency and the widest distribution, and it was the most ancestral haplotype. Hap_7 was shared by all the five of the Manila clam populations in China. These results indicate that there is a certain degree of gene exchange between the southern and northern Manila clam populations.

Manila clam breeding activities in China provide conditions for gene exchange between different populations. The main culture areas in China are in the north, but 90% of the breeding seeds come from Fujian and Guangxi in southern China. For instance, the two areas of Zhuanghe and Dandong in the North Yellow Sea annually produce about 1 million tons of Manila clams whose seeds are mainly from the south. Over a decade of continuous aquaculture, up to nearly 10 million tons of the Southern China-originated Manila clams were produced in the sea area of Northern China. The planktonic larvae of southern Manila clams have a strong ability to spread along the Yellow Sea and Bohai Sea offshore, resulting in a certain degree of genetic exchange with local northern indigenous populations. Similarly, Cordero et al.^6^ reported that the Japanese Manila clam population was introduced to the coast of British Columbia in North America in 1936 and then expanded to southern California in the United States after > 30 years of reproduction. Chiesa et al.^7^ reported that the Manila clams introduced to the European coasts of France, Britain, Italy, and Spain in 1972 exhibited a genetic introgression. In China, Manila clam farming using southern seedlings has caused the southern population to produce a large number of Manila clam larvae in the northern waters of China. The presence of these robust planktonic larvae together with the influence of the Yellow-Bohai Sea coastal current has promoted gene exchange between the Manila clam populations of South and North China.

### Genetic diversity of Manila clams

The levels of genetic diversity reflect the capability of a species to adapt to environmental changes. Species with high genetic variation have greater evolutionary potential and ecological adaptability. Conversely, species with low genetic variation generally have lower population resilience. In this study, we found that Hd of the seven populations was high and that the genetic diversity was higher, which showed that the COI gene of the Manila clam was highly polymorphic. Overall, the Manila clam is thought to have high genetic variation and diversity, which confers strong adaptability to the environmental parameters fluctuations and allows this species to distribute widely in coastal waters.

Our microsatellite data support this view. All 20 SSR loci were polymorphic, and the number of polymorphic alleles ranged from 4 to 10, with an average A_R_ of 3.22–6.15 per locus at the species level. This result was roughly in agreement with findings from previous reports^34,35^. This characteristic is expected considering the large population sizes of marine bivalves and the high mutation rates of microsatellites^10^.

PIC of the seven populations were was consistent with previous reports of the Manila clam populations from North America, Europe, and Korea^6,34,36^. Marine bivalves are commonly characterized by a high level of genetic diversity^10^. For the 20 pairs of primers, a positive value of Fis indicates a loss of heterozygotes, whereas a negative value indicates a loss of homozygotes. Therefore, these results indicate that most of the heterozygotes at the loci are missing, and this phenomenon may be caused by the Wahlund effect.

## Conclusions

In East Asia, the natural geographical isolation of the Yangtze River and the Yalu River divides the indigenous East Asian Manila clam populations into three geographic populations, the South China population, the North China population and the Japan-North Korea population. Large genetic differences among wild populations may be related to geographic isolation of these populations. The use of Manila clam seedlings from southern China for Aquaculture breeding in northern China, the coastal flow of the Yellow Sea, and the spread of planktonic clam larvae have likely promoted genetic exchange between South and North China populations. We found the genetic homogeneity between populations of Hokkaido and Sinuiju, due to Japan developed aquatic products on the west coast of Korea in 1910s.

Therefore, we propose to protect the germplasm resources of the indigenous populations in the north by creating a high-quality stock farm. Natural wild seedlings from the original seed farm could be systematically harvested and transferred to a different area for cultivation following protocols for sustainable utilization of Manila clam germplasm. The Laizhou population had the highest level of genetic diversity, which suggests that it has the greatest potential to adapt to future environmental changes. Therefore, this population is the most suitable for habitat protection and for in situ and off-site conservation and management strategies.

The results showed that the distribution curves of the mismatch were bimodal or multi-peak. Population mismatch indicated that population expansion of Manila clams did not occur recently. But Bayesian skyline method showed the population of Manila clams has undergone population expansion at the 12,500 years ago during the long evolutionary process, then the population turn to be stable after 11,000 years. In the future, a more precise study on the geographic population expansion of the Manila clams could be made through increasing the number of individuals from each geographic population or adding the data of simplified genome and genome re-sequencing.

## Supplementary Information

Supplementary Information.

## Data Availability

The datasets generated during and/or analysed during the current study are available from the corresponding author on reasonable request.
