# Supplementary material for: Population genetics of the Manila clam (Ruditapes philippinarum) in East Asia
Source: Sci Rep. 2020 Dec 14;10:21890. doi: 10.1038/s41598-020-78923-w (PMC7736867; doi:10.1038/s41598-020-78923-w)

**Supplementary information**

**Population genetics of the Manila clam (*Ruditapes philippinarum*) in East Asia**

**Yue Tan, Lei Fang, Ming Qiu, Zhongming Huo, Xiwu Yan**

**Contents:**

**- Supplementary Table S1**

**- Supplementary Table S2**

**- Supplementary Table S3**

**- Supplementary Table S4**

**-Supplementary Table S5**

**- Supplementary Figure S1**

**- Supplementary Figure S2**

**Table S1.** Characterization of 20 polymorphic microsatellite markers isolated from the Manila clam

| Locus | Repeat |  | Primer sequence | Ta°C | size |
| --- | --- | --- | --- | --- | --- |
|  | Motif |  |  |  |  |
| Rpg14883 | (ACATA)_4_ | F | AACCCGTAAGAATTATTTTGAACGAA | 62 | 174-215 |
|  |  | R | CACGACTGCTGTCAATTTATGAAGA |  |  |
| Rpg15289 | (TATGT)_4_ | F | AGTATTTCATGCATTGTAAATGGCTA | 62 | 152-179 |
|  |  | R | AATTACAGTTTTTCAATTGTTGACCA |  |  |
| Rpg15429 | (ATGTA)_4_ | F | GCAGTGATCATGGAAAGCTGTAAGT | 62 | 131-200 |
|  |  | R | GCATTGTTTGCAGAGTTACTGGGT |  |  |
| Rpg15656 | (ATACA)_6_ | F | TCGTGGTATCAACAATTTGAGCAT | 62 | 148-183 |
|  |  | R | TCACGTGAGTTCTGGTTGTCATTT |  |  |
| Rpg15246 | (TTATG)_4_ | F | TATCAATATTGGCAAAAAGGGGTG | 56 | 120-149 |
|  |  | R | CCACTACGCTATTACGCTGAACAA |  |  |
| Rpg15130 | (TAATG)_4_ | F | CACCACTTTCAGAGGTGAATACTGC | 56 | 146-176 |
|  |  | R | GGAGGATGGAACCTATCAGGTGAG |  |  |
| Rpg15387 | (TGTCG)_7_ | F | AAATTAGAGGCCCGTCTAGAACAT | 56 | 137-196 |
|  |  | R | GCAGATGTTAAAGCATAACATGACA |  |  |
| Rpg16260 | (CACAC)_4_ | F | CACACCACACCACACCACAC | 60 | 128-155 |
|  |  | R | CCAGTCATGTGGCAATCTTTATAGC |  |  |
| Rpg16726 | (GTGTA)_5_ | F | GTGTAGTGTAGTGTAGTGTAGTGTA | 60 | 117-178 |
|  |  | R | ACCCACTCCATTACACCTGACATC |  |  |
| Rpg16876 | (TTCTG)_4_ | F | TTCTGTTCTGTTCTGTTCTG | 60 | 128-145 |
|  |  | R | TGCGTGAAAACAAATATGTCCACT |  |  |
| Rpg16965 | (AGGTG)_4_ | F | AGGTGAGGTGAGGTGAGGTG | 60 | 113-133 |
|  |  | R | TGGCTAACCTTCGGTGTTACAAAC |  |  |
| Rpg17929 | (AAAAC)_4_ | F | AAAACAAAACAAAACAAAAC | 62 | 134-174 |
|  |  | R | AAATGCTAATGTAGAGAGAACCATTCAC |  |  |
| Rpg17997 | (TGCCC)_5_ | F | TGCCCTGCCCTGCCCTGCCCTGCCC | 62 | 133-173 |
|  |  | R | AATCAAATGATCACATACCCCTAATGT |  |  |
| Rpg17778 | (AGAAAG)_4_ | F | AGAAAGAGAAAGAGAAAGAGAAAG | 62 | 120-175 |
|  |  | R | ATACTTCAATGAAATGCGCAGACC |  |  |
| Rpg18096 | (AGAAC)_4_ | F | AGAACAGAACAGAACAGAAC | 62 | 150-178 |
|  |  | R | TTTCCCATATGACTACCCTTGCAT |  |  |
| Rpg16524 | (TGACCT)_4_ | F | TGACCTTGACCTTGACCTTGACCT | 56 | 141-161 |
|  |  | R | CAGATATCTTGTCCGGTCGGTAAC |  |  |
| Rpg15608 | (AGCAC)_6_ | F | CGATAGTGGAACATTCGACAGATG | 56 | 133-175 |
|  |  | R | TGTTGGAAACATACCTACTCACAATGA |  |  |
| Rpg16975 | (ATTTG)_4_ | F | ATTTGATTTGATTTGATTTG | 56 | 166-208 |
|  |  | R | GAGGGGAAGTGAGAAGCTGTTTAG |  |  |
| Rpg18002 | (AATTG)_4_ | F | AATTGAATTGAATTGAATTG | 62 | 140-169 |
|  |  | R | TAAACGACATTCTTTTGAACCCGT |  |  |
| Rpg18070 | (TGATA)_4_ | F | TGATATGATATGATATGATATGATA | 62 | 140-182 |
|  |  | R | ACGACAAATTGCCAAATGGTTACT |  |  |

**Table S2.** Haplotypes distributions of Ruditapes philippinarum

| Haplotype | CHI-LY(10) | CHI-FL(10) | CHI-SL(10) | CHI-GB(10) | CHI-TZ(10) | DPRK-S(10) | JAP-H(10) | Mean |
| --- | --- | --- | --- | --- | --- | --- | --- | --- |
| Hap1 | 1 |  |  |  |  |  |  | 1 |
| Hap2 | 1 |  |  |  |  |  |  | 1 |
| Hap3 | 1 |  |  |  |  |  | 2 | 3 |
| Hap4 | 1 |  |  |  |  |  |  | 1 |
| Hap5 | 1 |  |  |  |  |  |  | 1 |
| Hap6 | 1 |  |  |  |  |  |  | 1 |
| Hap7 | 2 | 2 | 2 | 1 | 4 |  |  | 11 |
| Hap8 | 1 |  |  |  |  |  |  | 1 |
| Hap9 | 1 |  | 1 |  | 1 |  |  | 3 |
| Hap10 |  | 4 | 1 |  |  |  |  | 5 |
| Hap11 |  | 1 |  |  |  |  |  | 1 |
| Hap12 |  | 1 |  |  |  |  |  | 1 |
| Hap13 |  | 1 |  |  |  |  |  | 1 |
| Hap14 |  | 1 |  |  |  |  |  | 1 |
| Hap15 |  |  | 1 |  |  |  |  | 1 |
| Hap16 |  |  | 1 |  |  |  |  | 1 |
| Hap17 |  |  | 1 |  |  |  |  | 1 |
| Hap18 |  |  | 1 |  |  |  |  | 1 |
| Hap19 |  |  | 1 |  |  |  |  | 1 |
| Hap20 |  |  | 1 |  | 2 |  |  | 3 |
| Hap21 |  |  |  | 2 |  |  |  | 2 |
| Hap22 |  |  |  | 2 |  |  |  | 2 |
| Hap23 |  |  |  | 2 |  |  |  | 2 |
| Hap24 |  |  |  | 1 |  |  |  | 1 |
| Hap25 |  |  |  | 1 |  |  |  | 1 |
| Hap26 |  |  |  | 1 |  |  |  | 1 |
| Hap27 |  |  |  |  | 1 |  |  | 1 |
| Hap28 |  |  |  |  | 1 |  |  | 1 |
| Hap29 |  |  |  |  | 1 |  |  | 1 |
| Hap30 |  |  |  |  |  | 1 |  | 1 |
| Hap31 |  |  |  |  |  | 4 | 4 | 8 |
| Hap32 |  |  |  |  |  | 1 |  | 1 |
| Hap33 |  |  |  |  |  | 1 |  | 1 |
| Hap34 |  |  |  |  |  | 1 |  | 1 |
| Hap35 |  |  |  |  |  | 1 |  | 1 |
| Hap36 |  |  |  |  |  | 1 |  | 1 |
| Hap37 |  |  |  |  |  |  | 1 | 1 |
| Hap38 |  |  |  |  |  |  | 1 | 1 |
| Hap39 |  |  |  |  |  |  | 1 | 1 |
| Hap40 |  |  |  |  |  |  | 1 | 1 |

**Table S3.** Genetic diversity of mtDNA COI gene of *Ruditapes philippinarum* among 7 different geographic populations

| Population | Number of haplotypes | Haplotypes ( number of individuals) | Haplotype diversity (Hd) | Nucleotide diversity (π) | Average number of nucleotide differences (K) |
| --- | --- | --- | --- | --- | --- |
| CHI-LY | 9 | H1(1) H2(4) H3(1) H4(1) H5(1) H6(1) H7(2) H8(1) H9(1) | 0.9778±0.054 | 0.00902±0.00096 | 6.178±2.517 |
| CHI-FL | 6 | H7(2) H10(4) H11(1) H12(1) H13(1) H14(1) | 0.8440±0.103 | 0.00509±0.00221 | 3.489±1.421 |
| CHI-SL | 9 | H7(2) H9(1) H10(1) H15(1) H16(1) H17(1) H18(1) H19(1) H20(1) | 0.9778±0.054 | 0.01446±0.00206 | 9.889±4.029 |
| CHI-GB | 7 | H7(1) H21(2) H22(2) H23(2) H24(1) H25(1) H26(1) | 0.9556±0.059 | 0.01278±0.00295 | 8.689±3.540 |
| CHI-TZ | 6 | H7(4) H9(1) H20(2) H27(1) H28(1) H29(1) | 0.8440±0.103 | 0.00918±0.00137 | 6.267±2.553 |
| JAP-H | 7 | H30(1) H31(4) H32(1) H33(1) H34(1) H35(1) H36(1) | 0.8440±0.103 | 0.00279±0.00071 | 1.911±0.779 |
| DPRK-S | 6 | H3(2) H31(4) H37(1) H38(1) H39(1) H40(1) | 0.8667±0.107 | 0.00794±0.00241 | 5.422±2.209 |
| Total | 40 | H1~H40 | 0.9565±0.014 | 0.01124±0.00079 | 7.606±2.609 |

**Table S4.** Genetic diversity of seven wild Manila clam populations. Number of alleles per locus (N*_A_*), allelic richness (A*_R_*), Shannon's Information index(*I*), expected heterozygosity (H*_E_*), observed heterozygosity (H*_O_*), inbreeding coefficient (F*_IS_*) are given for each population and locus. *Significant departure from Hardy-Weinberg equilibrium after Bonferoni correction (P < 0.01).

| Microsatellite loci | Populations | | | | | | |  |
| --- | --- | --- | --- | --- | --- | --- | --- | --- |
|  | CHI-YK | CHI-FL | CHI-SL | CHI-GB | CHI-TZ | JAP-H | DPRK-S | Mean |
| RPg14883 |  |  |  |  |  |  |  |  |
| *N*_A_ | 4.0000 | 5.0000 | 6.0000 | 5.0000 | 5.0000 | 5.0000 | 5.0000 | 5.0000 |
| *A*_R_ | 2.4260 | 3.2533 | 3.3625 | 2.7144 | 3.0254 | 3.7793 | 4.5872 | 3.3069 |
| *I* | 1.0113 | 1.3282 | 1.3975 | 1.1999 | 1.2908 | 1.4411 | 1.5579 | 1.3181 |
| *H*_E_ | 0.5937 | 0.7001 | 0.7097 | 0.6380 | 0.6786 | 0.7428 | 0.7899 | 0.6933 |
| *H*_O_ | 0.0800 | 0.1915 | 0.3800 | 0.5600 | 0.1892 | 0.1200 | 0.0400 | 0.2230 |
| *F*_IS_ | 0.8639* | 0.7235* | 0.4592* | 0.1134* | 0.7174* | 0.8368* | 0.9488* | 0.6661 |
| RPg15289 |  |  |  |  |  |  |  |  |
| *N*_A_ | 4.0000 | 4.0000 | 6.0000 | 4.0000 | 6.0000 | 5.0000 | 6.0000 | 5.0000 |
| *A*_R_ | 2.4534 | 2.9994 | 4.5620 | 2.8852 | 5.6570 | 3.5186 | 4.2662 | 3.7631 |
| *I* | 1.1138 | 1.2342 | 1.6111 | 1.1513 | 1.7594 | 1.4215 | 1.5884 | 1.4114 |
| *H*_E_ | 0.5984 | 0.6735 | 0.7887 | 0.6600 | 0.8345 | 0.7230 | 0.7733 | 0.7216 |
| *H*_O_ | 0.2800 | 0.0612 | 0.3000 | 0.3400 | 0.4054 | 0.2000 | 0.1400 | 0.2467 |
| *F*_IS_ | 0.5273* | 0.9082* | 0.6158* | 0.4796* | 0.5075* | 0.7206* | 0.8171* | 0.6537 |
| RPg15429 |  |  |  |  |  |  |  |  |
| *N*_A_ | 6.0000 | 9.0000 | 6.0000 | 10.0000 | 8.0000 | 7.0000 | 7.0000 | 7.5714 |
| *A*_R_ | 4.8900 | 6.4027 | 4.5662 | 7.0822 | 6.2943 | 4.4843 | 4.3719 | 5.4417 |
| *I* | 1.6618 | 2.0023 | 1.6261 | 2.0904 | 1.9236 | 1.6921 | 1.6282 | 1.8035 |
| *H*_E_ | 0.8037 | 0.8525 | 0.7889 | 0.8675 | 0.8526 | 0.7848 | 0.7794 | 0.8185 |
| *H*_O_ | 0.4082 | 0.2653 | 0.5600 | 0.9200 | 0.3514 | 0.2600 | 0.2708 | 0.4337 |
| *F*_IS_ | 0.4869* | 0.6856* | 0.2830* | -0.0713* | 0.5823* | 0.6654* | 0.6488* | 0.4687 |
| RPg15656 |  |  |  |  |  |  |  |  |
| *N*_A_ | 6.0000 | 6.0000 | 5.0000 | 6.0000 | 4.0000 | 5.0000 | 7.0000 | 5.5714 |
| *A*_R_ | 4.3934 | 4.1815 | 4.6948 | 4.9969 | 2.7217 | 3.6153 | 5.0556 | 4.2370 |
| *I* | 1.5749 | 1.5710 | 1.5771 | 1.6564 | 1.1660 | 1.4069 | 1.7451 | 1.5282 |
| *H*_E_ | 0.7803 | 0.7689 | 0.7949 | 0.8081 | 0.6412 | 0.7307 | 0.8103 | 0.7621 |
| *H*_O_ | 0.5306 | 0.2500 | 0.2800 | 0.1429 | 0.2703 | 0.1200 | 0.2400 | 0.2620 |
| *F*_IS_ | 0.3130* | 0.6714* | 0.6442* | 0.8214* | 0.5727* | 0.8341* | 0.7008* | 0.6511 |
| RPg15246 |  |  |  |  |  |  |  |  |
| *N*_A_ | 5.0000 | 6.0000 | 6.0000 | 5.0000 | 9.0000 | 6.0000 | 5.0000 | 6.0000 |
| *A*_R_ | 2.7732 | 4.3067 | 5.7937 | 4.8828 | 6.9865 | 5.1975 | 3.8256 | 4.8237 |
| *I* | 1.1834 | 1.6120 | 1.7738 | 1.5970 | 2.0557 | 1.7029 | 1.3917 | 1.6166 |
| *H*_E_ | 0.6459 | 0.7757 | 0.8358 | 0.8032 | 0.8689 | 0.8158 | 0.7461 | 0.7845 |
| *H*_O_ | 0.6600 | 0.4694 | 0.8200 | 0.5600 | 0.6111 | 0.8000 | 0.9400 | 0.6944 |
| *F*_IS_ | -0.0322 | 0.3887* | 0.0089* | 0.2958* | 0.2868* | 0.0094* | -0.2727* | 0.0978 |
| RPg15130 |  |  |  |  |  |  |  |  |
| *N*_A_ | 4.0000 | 5.0000 | 5.0000 | 4.0000 | 5.0000 | 5.0000 | 5.0000 | 4.7143 |
| *A*_R_ | 3.7453 | 4.1829 | 3.9936 | 3.4507 | 3.7809 | 4.3290 | 4.2481 | 3.9615 |
| *I* | 1.3519 | 1.4937 | 1.4609 | 1.2967 | 1.4171 | 1.5255 | 1.5137 | 1.4371 |
| *H*_E_ | 0.7404 | 0.7688 | 0.7572 | 0.7174 | 0.7462 | 0.7768 | 0.7723 | 0.7542 |
| *H*_O_ | 0.9800 | 1.0000 | 0.9000 | 0.8800 | 0.5714 | 0.7000 | 0.7200 | 0.8216 |
| *F*_IS_ | -0.3370* | -0.3142* | -0.2006* | -0.2391* | 0.2231* | 0.0897 | 0.0583* | -0.1028 |
| RPg15387 |  |  |  |  |  |  |  |  |
| *N*_A_ | 5.0000 | 6.0000 | 8.0000 | 6.0000 | 5.0000 | 9.0000 | 9.0000 | 6.8571 |
| *A*_R_ | 3.8610 | 3.5888 | 6.5789 | 4.4843 | 2.0774 | 4.3440 | 6.8027 | 4.5339 |
| *I* | 1.4300 | 1.4791 | 1.9671 | 1.5859 | 1.0174 | 1.6914 | 2.0164 | 1.5982 |
| *H*_E_ | 0.7485 | 0.7289 | 0.8566 | 0.7848 | 0.5257 | 0.7776 | 0.8616 | 0.7548 |
| *H*_O_ | 0.3800 | 0.1042 | 0.7400 | 0.3600 | 0.0541 | 0.6000 | 0.7600 | 0.4283 |
| *F*_IS_ | 0.4872* | 0.8556* | 0.1274 | 0.5367* | 0.8958* | 0.2206* | 0.1090* | 0.4618 |
| RPg16260 |  |  |  |  |  |  |  |  |
| *N*_A_ | 5.0000 | 5.0000 | 4.0000 | 5.0000 | 4.0000 | 5.0000 | 5.0000 | 4.7143 |
| *A*_R_ | 4.0750 | 4.2952 | 3.0544 | 4.3706 | 2.6151 | 3.6023 | 2.6427 | 3.5222 |
| *I* | 1.4859 | 1.5275 | 1.2252 | 1.5205 | 1.1126 | 1.4233 | 1.1346 | 1.3471 |
| *H*_E_ | 0.7622 | 0.7751 | 0.6794 | 0.7790 | 0.6261 | 0.7297 | 0.6279 | 0.7113 |
| *H*_O_ | 0.5800 | 0.7755 | 0.4800 | 0.3000 | 0.0811 | 0.1600 | 0.0400 | 0.3452 |
| *F*_IS_ | 0.2314* | -0.0109* | 0.2864* | 0.6110* | 0.8687* | 0.7785* | 0.9356* | 0.5287 |
| RPg16726 |  |  |  |  |  |  |  |  |
| *N*_A_ | 5.0000 | 5.0000 | 5.0000 | 5.0000 | 5.0000 | 6.0000 | 8.0000 | 5.5714 |
| *A*_R_ | 3.7793 | 4.3379 | 3.2196 | 4.3440 | 3.3228 | 3.8202 | 3.7538 | 3.7968 |
| *I* | 1.4630 | 1.5266 | 1.3600 | 1.5321 | 1.3229 | 1.5356 | 1.5720 | 1.4732 |
| *H*_E_ | 0.7428 | 0.7774 | 0.6964 | 0.7776 | 0.7086 | 0.7458 | 0.7410 | 0.7414 |
| *H*_O_ | 0.4400 | 0.6735 | 0.5200 | 0.2200 | 0.3784 | 0.5510 | 0.2600 | 0.4347 |
| *F*_IS_ | 0.4017* | 0.1248* | 0.2457* | 0.7142* | 0.4587* | 0.2536* | 0.6456* | 0.4063 |
| RPg16876 |  |  |  |  |  |  |  |  |
| *N*_A_ | 4.0000 | 5.0000 | 5.0000 | 5.0000 | 3.0000 | 5.0000 | 5.0000 | 4.5714 |
| *A*_R_ | 3.1133 | 3.6434 | 3.3311 | 3.4924 | 1.8475 | 3.5613 | 3.5997 | 3.2270 |
| *I* | 1.2343 | 1.4276 | 1.3393 | 1.3635 | 0.8034 | 1.4145 | 1.4114 | 1.2849 |
| *H*_E_ | 0.6857 | 0.7330 | 0.7069 | 0.7210 | 0.4650 | 0.7265 | 0.7295 | 0.6811 |
| *H*_O_ | 0.2600 | 0.7551 | 0.4800 | 0.1633 | 0.0000 | 0.5800 | 0.5600 | 0.3998 |
| *F*_IS_ | 0.6170* | -0.0408* | 0.3141* | 0.7712* | 1.0000* | 0.1935 | 0.2246* | 0.4399 |
| RPg16965 |  |  |  |  |  |  |  |  |
| *N*_A_ | 4.0000 | 4.0000 | 5.0000 | 4.0000 | 4.0000 | 4.0000 | 5.0000 | 4.2857 |
| *A*_R_ | 3.3535 | 2.9050 | 3.9124 | 3.1270 | 2.4914 | 3.3535 | 4.0193 | 3.3089 |
| *I* | 1.2835 | 1.1841 | 1.4664 | 1.2411 | 1.0314 | 1.2800 | 1.4614 | 1.2783 |
| *H*_E_ | 0.7089 | 0.6625 | 0.7519 | 0.6871 | 0.6068 | 0.7089 | 0.7588 | 0.6978 |
| *H*_O_ | 0.5800 | 0.3878 | 0.8000 | 0.3000 | 0.4054 | 0.3800 | 0.2000 | 0.4362 |
| *F*_IS_ | 0.1736* | 0.4087* | -0.0747 | 0.5590* | 0.3228* | 0.4585* | 0.7338* | 0.3688 |
| RPg17929 |  |  |  |  |  |  |  |  |
| *N*_A_ | 5.0000 | 6.0000 | 4.0000 | 4.0000 | 5.0000 | 5.0000 | 6.0000 | 5.0000 |
| *A*_R_ | 3.2468 | 3.0297 | 2.6896 | 3.1908 | 4.5633 | 2.8986 | 3.9002 | 3.3599 |
| *I* | 1.3477 | 1.3072 | 1.1413 | 1.2258 | 1.5521 | 1.2483 | 1.5442 | 1.3381 |
| *H*_E_ | 0.6990 | 0.6768 | 0.6345 | 0.6935 | 0.7916 | 0.6616 | 0.7511 | 0.7012 |
| *H*_O_ | 0.4200 | 0.5714 | 0.2400 | 0.1200 | 0.2162 | 0.1800 | 0.2400 | 0.2839 |
| *F*_IS_ | 0.3931* | 0.1470 | 0.6180* | 0.8252* | 0.7231* | 0.7252* | 0.6772* | 0.5870 |
| RPg17997 |  |  |  |  |  |  |  |  |
| *N*_A_ | 5.0000 | 6.0000 | 7.0000 | 4.0000 | 6.0000 | 5.0000 | 8.0000 | 5.8571 |
| *A*_R_ | 3.2830 | 4.8603 | 3.8880 | 3.1309 | 4.6172 | 2.9851 | 3.2425 | 3.7153 |
| *I* | 1.3059 | 1.6728 | 1.5812 | 1.2179 | 1.6437 | 1.2102 | 1.5548 | 1.4552 |
| *H*_E_ | 0.7024 | 0.8024 | 0.7503 | 0.6875 | 0.7942 | 0.6717 | 0.6986 | 0.7296 |
| *H*_O_ | 0.3800 | 0.4490 | 0.5600 | 0.1400 | 0.3243 | 0.1200 | 0.4200 | 0.3419 |
| *F*_IS_ | 0.4536* | 0.4347* | 0.2461* | 0.7943* | 0.5860* | 0.8195* | 0.3927* | 0.5324 |
| RPg17778 |  |  |  |  |  |  |  |  |
| *N*_A_ | 5.0000 | 7.0000 | 6.0000 | 3.0000 | 3.0000 | 5.0000 | 5.0000 | 4.8571 |
| *A*_R_ | 2.4414 | 3.9264 | 4.7847 | 2.5471 | 2.4578 | 4.1667 | 3.8670 | 3.4559 |
| *I* | 1.0510 | 1.5501 | 1.6567 | 0.9973 | 0.9882 | 1.5016 | 1.4293 | 1.3106 |
| *H*_E_ | 0.5964 | 0.7530 | 0.7990 | 0.6135 | 0.6013 | 0.7677 | 0.7489 | 0.6971 |
| *H*_O_ | 0.2600 | 0.7551 | 0.4800 | 0.2200 | 0.0000 | 0.8600 | 0.6600 | 0.4622 |
| *F*_IS_ | 0.5596* | -0.0131* | 0.3932* | 0.6378* | 1.0000* | -0.1316* | 0.1098* | 0.3651 |
| RPg18096 |  |  |  |  |  |  |  |  |
| *N*_A_ | 4.0000 | 4.0000 | 6.0000 | 4.0000 | 5.0000 | 6.0000 | 6.0000 | 5.0000 |
| *A*_R_ | 3.3852 | 2.9919 | 3.0157 | 3.5486 | 4.0124 | 4.3178 | 3.5613 | 3.5476 |
| *I* | 1.2779 | 1.1722 | 1.2915 | 1.3108 | 1.4938 | 1.5749 | 1.3856 | 1.3581 |
| *H*_E_ | 0.7117 | 0.6726 | 0.6752 | 0.7255 | 0.7613 | 0.7762 | 0.7265 | 0.7213 |
| *H*_O_ | 0.2800 | 0.5306 | 0.2200 | 0.1400 | 0.2222 | 0.2800 | 0.2000 | 0.2675 |
| *F*_IS_ | 0.6026* | 0.2030* | 0.6709* | 0.8051* | 0.7040* | 0.6356* | 0.7219* | 0.6204 |
| RPg16524 |  |  |  |  |  |  |  |  |
| *N*_A_ | 4.0000 | 4.0000 | 4.0000 | 3.0000 | 6.0000 | 4.0000 | 4.0000 | 4.1429 |
| *A*_R_ | 2.8265 | 2.8065 | 2.7594 | 2.6610 | 4.4520 | 2.9976 | 2.7367 | 3.0342 |
| *I* | 1.1417 | 1.1687 | 1.1404 | 1.0381 | 1.5824 | 1.1843 | 1.1237 | 1.1970 |
| *H*_E_ | 0.6527 | 0.6503 | 0.6440 | 0.6305 | 0.7860 | 0.6731 | 0.6410 | 0.6682 |
| *H*_O_ | 0.4800 | 0.2653 | 0.4600 | 0.3200 | 0.2162 | 0.0400 | 0.1200 | 0.2716 |
| *F*_IS_ | 0.2572* | 0.5878* | 0.2785* | 0.4873* | 0.7211* | 0.9400* | 0.8109* | 0.5833 |
| RPg15608 |  |  |  |  |  |  |  |  |
| *N*_A_ | 7.0000 | 7.0000 | 8.0000 | 8.0000 | 6.0000 | 8.0000 | 12.0000 | 8.0000 |
| *A*_R_ | 5.6818 | 5.4630 | 7.1633 | 6.1652 | 4.7647 | 5.5371 | 8.3333 | 6.1583 |
| *I* | 1.8227 | 1.8065 | 2.0220 | 1.9346 | 1.6398 | 1.8438 | 2.2294 | 1.8998 |
| *H*_E_ | 0.8323 | 0.8254 | 0.8691 | 0.8463 | 0.8013 | 0.8277 | 0.8889 | 0.8416 |
| *H*_O_ | 0.5200 | 0.5918 | 0.6600 | 0.3200 | 0.4722 | 0.5800 | 0.5600 | 0.5291 |
| *F*_IS_ | 0.3689* | 0.2756* | 0.2329* | 0.6180* | 0.4023* | 0.2922* | 0.3636* | 0.3648 |
| RPg16975 |  |  |  |  |  |  |  |  |
| *N*_A_ | 5.0000 | 6.0000 | 5.0000 | 4.0000 | 3.0000 | 5.0000 | 6.0000 | 4.8571 |
| *A*_R_ | 3.7092 | 3.5413 | 3.6311 | 1.7864 | 1.8922 | 3.8850 | 4.3066 | 3.2503 |
| *I* | 1.4423 | 1.4297 | 1.4105 | 0.8658 | 0.7636 | 1.4448 | 1.5475 | 1.2720 |
| *H*_E_ | 0.7378 | 0.7250 | 0.7319 | 0.4446 | 0.4780 | 0.7501 | 0.7756 | 0.6633 |
| *H*_O_ | 0.8800 | 0.7347 | 0.6600 | 0.5000 | 0.3243 | 0.9800 | 0.8800 | 0.7084 |
| *F*_IS_ | -0.2048* | -0.0238 | 0.0892 | -0.1358 | 0.3122 | -0.3197* | -0.1461 | -0.0613 |
| Rpg18002 |  |  |  |  |  |  |  |  |
| *N*_A_ | 4.0000 | 5.0000 | 5.0000 | 4.0000 | 9.0000 | 3.0000 | 5.0000 | 5.0000 |
| *A*_R_ | 2.9155 | 2.5529 | 3.6364 | 2.5543 | 6.8622 | 2.3223 | 3.6049 | 3.4926 |
| *I* | 1.1719 | 1.0923 | 1.3916 | 1.0628 | 2.0125 | 0.9240 | 1.4122 | 1.2953 |
| *H*_E_ | 0.6636 | 0.6146 | 0.7323 | 0.6149 | 0.8660 | 0.5752 | 0.7299 | 0.6852 |
| *H*_O_ | 0.4800 | 0.4898 | 0.3000 | 0.0625 | 0.3243 | 0.1600 | 0.0200 | 0.2624 |
| *F*_IS_ | 0.2694* | 0.1948 | 0.5862* | 0.8973* | 0.6204* | 0.7190* | 0.9723 | 0.6085 |
| Rpg18070 |  |  |  |  |  |  |  |  |
| *N*_A_ | 4.0000 | 5.0000 | 8.0000 | 5.0000 | 6.0000 | 4.0000 | 5.0000 | 5.2857 |
| *A*_R_ | 2.5641 | 2.8789 | 7.5188 | 3.6024 | 3.4012 | 3.6853 | 3.6550 | 3.9008 |
| *I* | 1.0654 | 1.2841 | 2.0487 | 1.3912 | 1.4038 | 1.3426 | 1.4141 | 1.4214 |
| *H*_E_ | 0.6162 | 0.6594 | 0.8758 | 0.7299 | 0.7157 | 0.7362 | 0.7337 | 0.7238 |
| *H*_O_ | 0.4200 | 0.3878 | 0.6000 | 0.3061 | 0.1892 | 0.1020 | 0.0000 | 0.2864 |
| *F*_IS_ | 0.3115* | 0.4059* | 0.3080* | 0.5762* | 0.7320* | 0.8600 | 1.0000* | 0.5991 |
| Mean |  |  |  |  |  |  |  |  |
| *N*_A_ | 4.7500 | 5.5000 | 5.7000 | 4.9000 | 5.3500 | 5.3500 | 6.2000 | 5.3929 |
| *A*_R_ | 3.4458 | 3.8074 | 4.3078 | 3.7509 | 3.8921 | 3.8200 | 4.2190 | 3.8919 |
| *I* | 1.3210 | 1.4435 | 1.5244 | 1.3640 | 1.3990 | 1.4405 | 1.5331 | 1.4322 |
| *H*_E_ | 0.7011 | 0.7298 | 0.7539 | 0.7115 | 0.7075 | 0.7351 | 0.7542 | 0.7276 |
| *H*_O_ | 0.4649 | 0.4854 | 0.5220 | 0.3437 | 0.2803 | 0.3887 | 0.3635 | 0.4069 |

**Table S5.** Polymorphism Information Content (PIC) of 20 microsatellite loci in seven different geographic populations

|  | CHI-YK | CHI-FL | CHI-SL | CHI-GB | CHI-TZ | JAP-H | DPRK-S | Mean |
| --- | --- | --- | --- | --- | --- | --- | --- | --- |
| RPg14883 | 0.51788 | 0.63993 | 0.65211 | 0.57283 | 0.62095 | 0.69220 | 0.74620 | 0.63459 |
| RPg15289 | 0.79818 | 0.61679 | 0.74771 | 0.58809 | 0.54884 | 0.67586 | 0.73019 | 0.67224 |
| RPg15429 | 0.76424 | 0.82547 | 0.74937 | 0.84336 | 0.82098 | 0.74833 | 0.73609 | 0.78398 |
| RPg15656 | 0.73608 | 0.72556 | 0.75296 | 0.76907 | 0.57938 | 0.67631 | 0.77472 | 0.71630 |
| RPg15246 | 0.57772 | 0.73450 | 0.80324 | 0.76235 | 0.84076 | 0.77923 | 0.69040 | 0.74117 |
| RPg15130 | 0.68378 | 0.72059 | 0.70709 | 0.65552 | 0.68967 | 0.73066 | 0.72566 | 0.70185 |
| RPg15387 | 0.69562 | 0.68330 | 0.82964 | 0.74088 | 0.48013 | 0.73051 | 0.83584 | 0.71370 |
| RPg16260 | 0.71413 | 0.73030 | 0.61335 | 0.73237 | 0.55211 | 0.68065 | 0.54706 | 0.65285 |
| RPg16726 | 0.69692 | 0.73125 | 0.64592 | 0.73249 | 0.64784 | 0.70514 | 0.69599 | 0.69365 |
| RPg16876 | 0.61947 | 0.68088 | 0.64896 | 0.66552 | 0.41100 | 0.67440 | 0.67563 | 0.62512 |
| RPg16965 | 0.64759 | 0.59064 | 0.70223 | 0.62522 | 0.52602 | 0.64702 | 0.70806 | 0.63525 |
| RPg17929 | 0.64298 | 0.61134 | 0.57282 | 0.62442 | 0.74449 | 0.59851 | 0.70773 | 0.64318 |
| RPg17997 | 0.64195 | 0.76413 | 0.70982 | 0.61959 | 0.75202 | 0.60006 | 0.66497 | 0.67894 |
| RPg17778 | 0.50562 | 0.70552 | 0.75965 | 0.52558 | 0.52150 | 0.71991 | 0.69574 | 0.63336 |
| RPg18096 | 0.64756 | 0.60101 | 0.61483 | 0.66416 | 0.71264 | 0.73154 | 0.66990 | 0.66309 |
| RPg16524 | 0.58220 | 0.58571 | 0.57597 | 0.55359 | 0.74012 | 0.60304 | 0.57103 | 0.60167 |
| RPg15608 | 0.80115 | 0.79321 | 0.84442 | 0.81847 | 0.75813 | 0.79668 | 0.85213 | 0.80917 |
| RPg16975 | 0.68868 | 0.67065 | 0.67784 | 0.41115 | 0.39222 | 0.69778 | 0.72898 | 0.60961 |
| RPg18002 | 0.59552 | 0.52919 | 0.67506 | 0.52857 | 0.83718 | 0.48030 | 0.67646 | 0.61747 |
| RPg18070 | 0.53444 | 0.60857 | 0.86802 | 0.67345 | 0.65574 | 0.67886 | 0.68227 | 0.67162 |
| Mean | 0.65459 | 0.67743 | 0.70755 | 0.65533 | 0.64159 | 0.68235 | 0.70575 |  |

**Figure S1** Bayesian model-based cluster analysis of individual genotype at COI locus in 7 populations of Manila clam. Diagram of posterier of the COI data according to Pritchard et al. for k=2 to k=4.


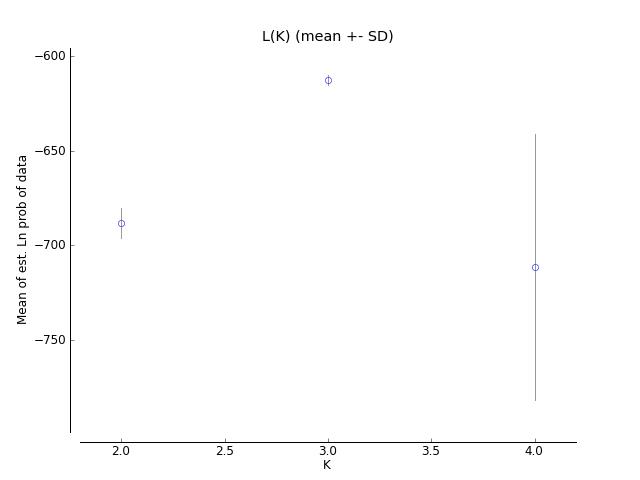


**Figure S2.** Pairwise comparisons of differences in Manila clams from the 7 populations based on the COI gene. Blue is the observed value, and the dotted red line with a circle is the expected value.


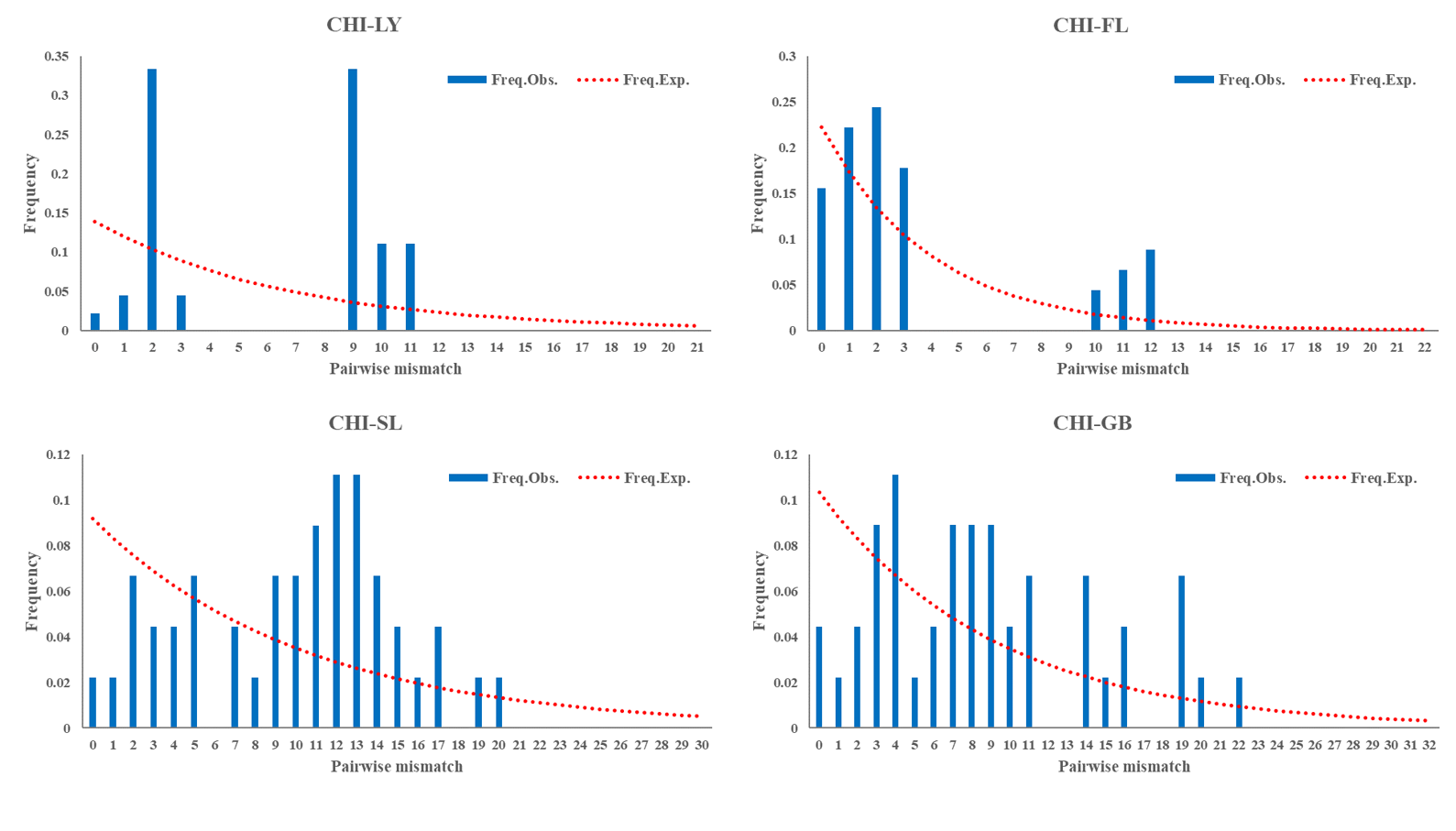


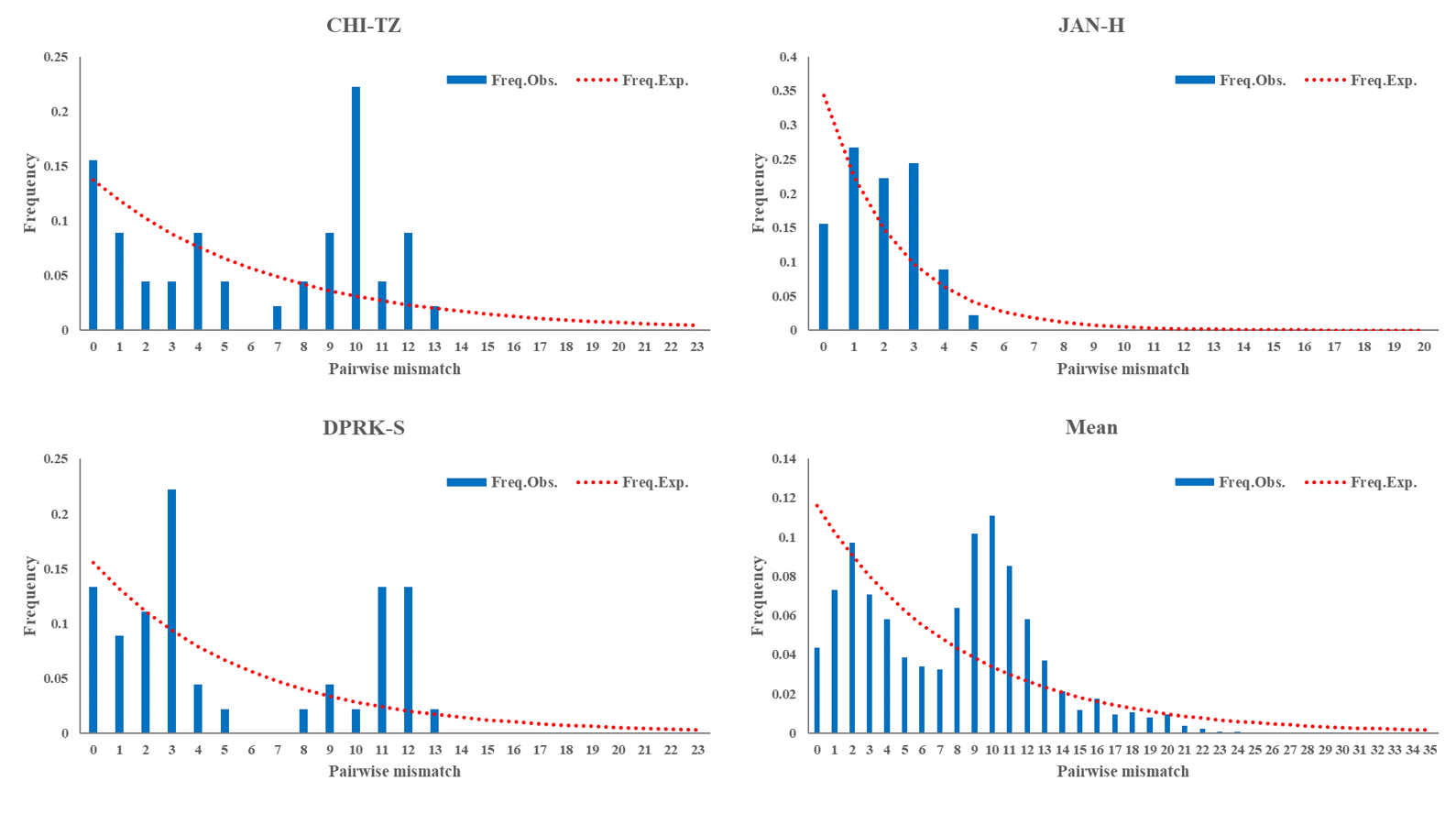

Supplement: Supplementary file 1 — Supplementary Information. [file 41598_2020_78923_MOESM1_ESM.docx]
